# School-Aged Outcomes After Neonatal Arterial Ischemic Stroke

**DOI:** 10.15844/pedneurbriefs-30-2-7

**Published:** 2016-02

**Authors:** Theresa Czech, Andrea C. Pardo

**Affiliations:** 1 Division of Neurology, Ann & Robert H. Lurie Children's Hospital of Chicago, Chicago, IL; 2 Departments of Pediatrics and Neurology, Northwestern University Feinberg School of Medicine, Chicago, IL

**Keywords:** Neonatal Arterial Ischemic Stroke, Survivor Outcomes, Epilepsy

## Abstract

Investigators from the Accident Vasculaire Cérébral de nouveau-né (AVCnn) Study Group, a multicenter registry in France, examined outcomes at 7 years of age in children previously identified with neonatal arterial ischemic stroke (NAIS).

Investigators from the Accident Vasculaire Cérébral de nouveau-né (AVCnn) Study Group, a multicenter registry in France, examined outcomes at 7 years of age in children previously identified with neonatal arterial ischemic stroke (NAIS). 100 patients were enrolled in this cohort, 80 patients completed at least part of the total assessment. Investigators obtained the patient's medical history, assessment of his/her developmental profile, and parental opinions on their child's medical sequelae from NAIS. Outcomes studied were presence of epilepsy, academic performance, presence and severity of cerebral palsy, presence of intellectual disability, presence of impaired spoken language, co-occurrence of deficits and parental opinion of outcomes. Impaired language was the most common problem, affecting 49% of children studied. The prevalence of the other outcomes was as follows: cerebral palsy 32%, low academic skills 28%, active epilepsy 11% and global intellectual impairment 8%. The investigators conclude that 59% of the patients in this cohort had any type of disability and that co-occurrence of disabilities is high. The investigators found a higher prevalence of speech impairment in comparison with other previously reported studies. This cohort presents a cross section of outcomes of school children presenting with NAIS in the neonatal period. [1]

COMMENTARY. Neonatal arterial ischemic stroke (NAIS) is by definition a symptomatic arterial ischemic infarct which presents within the first 28 days of life [2]. NAIS affects approximately 1 in 1600 to 1 in 5000 neonates [3]. It is likely that the total incidence of perinatal stroke is much higher than these quoted statistics since some children who are asymptomatic in the newborn period are later identified as having a presumed neonatal stroke by imaging when they present with early handedness, hemiplegia, epilepsy, or learning difficulties. The evolution of imaging findings and the variability of presenting symptoms makes it more difficult to study those children with presumed perinatal stroke cohort as compared to those identified with NAIS. Children with NAIS have been previously identified as being at risk for future development of motor deficits, cognitive disorders, and epilepsy [3]. This current study adds information on the prevalence of these issues at age 7 years in children with NAIS. While the overall occurrence of outcomes such has cerebral palsy, epilepsy, and intellectual impairment was similar to that reported in previous studies [3], this study explored in more detail the severity of impairment in each case. It is interesting to see that although many children have disabilities, most cases are mild. It is encouraging that all patients in this cohort are able to walk independently and only 8% have global intellectual disability. Previously reported data have shown an association between mixed infarctions and cerebrospinal tract involvement with worse motor outcomes [4]. This study adds important information that may be provided to caretakers regarding the long term outcome of infants presenting with NAIS. Additional studies would be needed to determine differences in long-term outcomes between children with a neonatal presentation and those with later presentation.

## References

[1] Chabrier S, Peyric E, Drutel L, Deron J, Kossorotoff M, Dinomais M (2016). Multimodal Outcome at 7 Years of Age after Neonatal Arterial Ischemic Stroke. J Pediatr.

[2] Raju TN, Nelson KB, Ferriero D, Lynch JK, NICHD-NINDS Perinatal Stroke Workshop Participants (2007). Ischemic perinatal stroke: summary of a workshop sponsored by the National Institute of Child Health and Human Development and the National Institute of Neurological Disorders and Stroke. Pediatrics.

[3] Lynch JK (2009). Epidemiology and classification of perinatal stroke. Semin Fetal Neonatal Med.

[4] Husson B, Hertz-Pannier L, Renaud C, Allard D, Presles E, Landrieu P, AVCnn Group (2010). Motor outcomes after neonatal arterial ischemic stroke related to early MRI data in a prospective study. Pediatrics.

